# The genus *Aspidimerus* Mulsant, 1850 (Coleoptera, Coccinellidae) from China, with descriptions of two new species

**DOI:** 10.3897/zookeys.348.5746

**Published:** 2013-11-08

**Authors:** Lizhi Huo, Xingmin Wang, Xiaosheng Chen, Shunxiang Ren

**Affiliations:** 1 Engineering Research Center of Biological Control, Ministry of Education; College of Natural Resources and Environment, South China Agricultural University, Guangzhou 510642, China

**Keywords:** Coleoptera, Coccinellidae, Aspidimerini, *Aspidimerus*, *Pseudaspidimerus*, new species, China

## Abstract

Chinese members of the genus *Aspidimerus* Mulsant, 1850 are reviewed. Ten species are recognized, including two new species: *A. zhenkangicus* Huo & Ren, **sp. n.** and *A. menglensis* Huo & Ren, **sp. n.** *A. kabakovi* Hoàng is recorded from China for the first time. *A. blandus* (Mader, 1954) is recognized as synonymous with *A. ruficrus* Gorham, 1895 **(syn. n.)**. *Aspidimerus rectangulatus* Kuznetsov & Pang, 1991 and *A. serratus* Kuznetsov & Pang, 1991 are transferred to the genus *Pseudaspidimerus* Kapur, 1948 **(comb. n.)**. All species from China are described and illustrated. Distribution maps of the Chinese species, a key and a catalogue of all known *Aspidimerus* are provided.

## Introduction

The genus *Aspidimerus* Mulsant, 1850 was erected with *Aspidimerusspencii* from India as the type species by monotypy. Concurrently, another genus *Cryptogonus* Mulsant was erected with *Cryptogonusorbiculus* (Gyllenhal, 1808) as the type species, also by monotypy. At the time of erecting these two genera, Mulsant had only one species for each genus, and separated them by the structure of the prosternum and the labrum which was covered or not by the clypeus. Chapuis (1876) and Weise (1885) considered these characters unreliable and the separation unwarranted as more species became known, and synonymised these two genera: Chapuis kept the name *Cryptogonus* whilst Weise preferred to retain *Aspidimerus*. After examining a large number of specimens, Weise (1900) considered that *Aspidimerus* and *Cryptogonus* were evidently different, based on the structure of male genitalia and consequently revived *Cryptogonus*,but separated them from the Scymnini, and assigned them to the present tribe Aspidimerini. Kapur (1948) revised the tribe Aspidimerini and erected two new genera: *Acarinus* and *Pseudaspidimerus*.

In the revision of the tribe Aspidimerini (Kapur, 1948), only three species of *Aspidimerus* were included: *Aspidimerus spencii* Mulsant, 1850, *Aspidimerus ruficrus* Gorham, 1895 and *Aspidimerus birmanicus* (Gorham, 1895). But Kapur did not study *Aspidimerus nigrovittatus* Motschulsky, 1866 nor *Aspidimerus mouhoti* Crotch, 1874, which were listed by Korschefsky (1931) in the Coleopterorum Catalogus. Subsequently, Sasaji (1968) described two species *Aspidimerus esakii* and *Aspidimerus matsumurai* from Taiwan, China. Pang and Mao (1979) added another two species *Aspidimerus sexmaculatus* and *Aspidimerus decemmaculatus* from Yunnan, China. Hoàng (1982) described four species *Aspidimerus laokayensis*, *Aspidimerus dongpaoensis*, *Aspidimerus chapaensis* and *Aspidimerus kabakovi* from Vietnam. Yu and Li (2004) added *Aspidimerus guangxiensis* to the *Aspidimerus* fauna from Guangxi, China.

Recently, Kovář (2007) transferred *Cryptogonus nigritus* Pang & Mao, 1979 and *Cryptogonus blandus* Mader, 1954 to *Aspidimerus*: *Aspidimerus nigritus* (Pang & Mao, 1979) and *Aspidimerusblandus* (Mader, 1954). *Aspidimerus dongpaoensis* Hoàng, 1982 was synonymized with *Aspidimerus nigritus* (Pang & Mao, 1979) and *Aspidimerus sexmaculatus* Pang & Mao, 1979 with *Aspidimerus mouhoti* Crotch, 1874. After examination of the specimens of *Aspidimerus blandus* (Mader, 1954) collected from the type locality, we found that the characters of the adult, including the male genitalia, perfectly agreed with the descriptions and illustrations of *Aspidimerus ruficrus* Gorham, 1895 given by Kapur (1948). Therefore, we consider *Aspidimerus blandus* (Mader, 1954) a junior synonym of *Aspidimerus ruficrus* Gorham, 1895.

Additionally, Kuznetsov and Pang (1991) described two species *Aspidimerus rectangulatus* and *Aspidimerus serratus* from Vietnam. Examination of the type series showed that the characters of the adults, including the male genitalia, are in perfect agreement with the diagnosis of *Pseudaspidimerus* Kapur, 1948. Therefore, these two species are transferred to the genus *Pseudaspidimerus*: *Pseudaspidimerus rectangulatus* (Kuznetsov & Pang, 1991) (comb. n.) and *Pseudaspidimerus serratus* (Kuznetsov & Pang, 1991) (comb. n.).

Until now, thirteen species of *Aspidimerus* have been described, all occurring in the Oriental Region. In this paper, ten species of *Aspidimerus* from China are revised, including two new species: *Aspidimerus zhenkangicus* Huo & Ren, sp. n. and *Aspidimerus menglensis* Huo & Ren, sp. n. *Aspidimerus kabakovi* Hoàng is recorded from China for the first time. Diagnoses, detailed descriptions, colored illustrations and distribution maps are provided for each species. A key and catalogue of all known species are also provided.

## Materials and methods

The specimens examined were collected and preserved in 90% ethanol. External morphology was observed with a dissecting stereo microscope (SteREO Discovery V20, Zeiss). The measurements were made with an ocular micrometer: total length, from apical margin of clypeus to apex of elytra (TL); total width, across both elytra at widest part (TW=EW); height, through the highest point of elytra to metaventrite (TH); head width, including eyes (HW); pronotal length, from the middle of anterior margin to the base of pronotum (PL); pronotal width at widest part (PW); elytral length, along the suture, from the apex to the base including the scutellum (EL). Male and female genitalia were dissected, cleared in a 10% solution of NaOH by boiling for several minutes, and examined with an Olympus BX51 compound microscope.

Images were photographed with digital cameras (AxioCam HRc and Coolsnap–Pro*cf* & CRI Micro*Color), connected to the dissecting microscope. The software AxioVision Rel. 4.8 and Image–Pro Plus 5.1 were used to capture images from both cameras, and photos were cleaned up and laid out in plates with Adobe Photoshop CS5.

Terminology follows Ślipiński (2007) and Ślipiński and Tomaszewska (2010). Specimens used in this study are deposited in the Department of Entomology, South China Agriculture University, Guangzhou, China (SCAU) and Institute of Zoology, Chinese Academy of Sciences (IOZ).

## Taxonomy

### Genus Aspidimerus

Mulsant, 1850

http://species-id.net/wiki/Aspidimerus

Figs 1–11

Aspidimerus Mulsant, 1850: 944. Type species: *Aspidimerusspencii* Mulsant, 1850, by monotypy.

#### Diagnosis.

**Figures 1–11.**
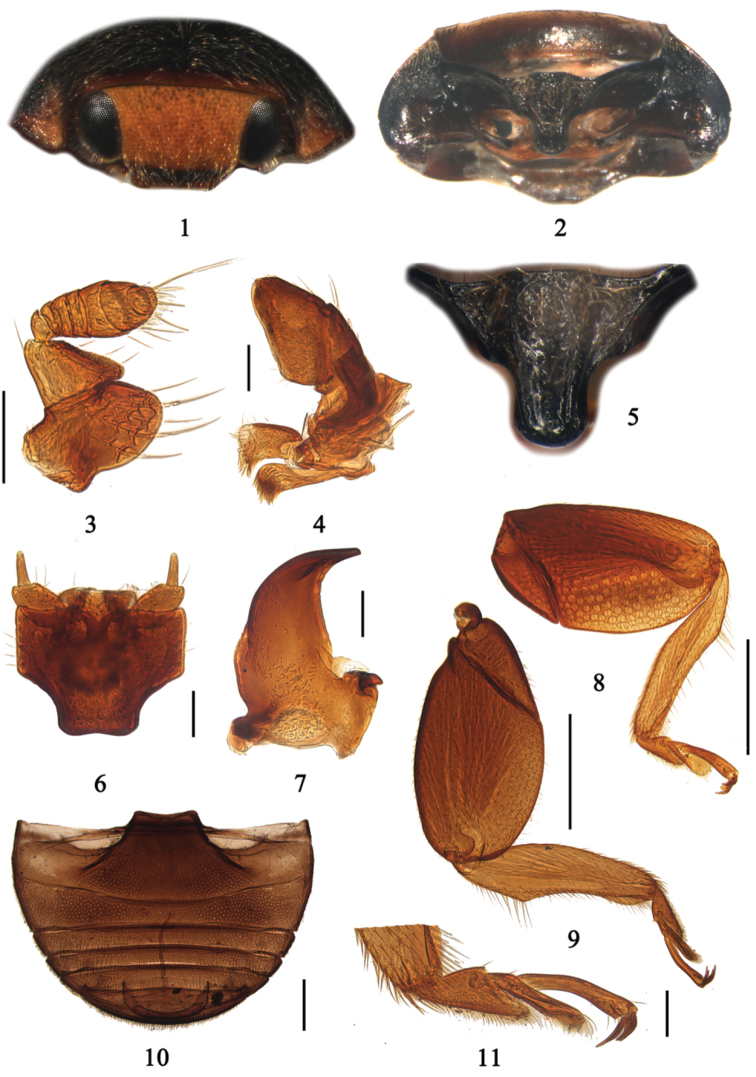
*Aspidimerus matsumurai* Sasaji, 1986. **1** head, frontal view **2** prothorax, ventral view **3** antenna **4** maxilla **5** prosternal process **6** labium **7** mandible **8** front leg **9** hind leg **10** abdomen **11** tarsi. Scale bars: Figures **1–7, 11**: 0.1mm; Figures **8–10**: 0.5mm.

*Aspidimerus* is closely related to *Cryptogonus* Mulsant. However, it can be easily distinguished from the latter as follows: prosternum T-shaped, evenly convex (Fig. 2), prosternal lines as wide apart as the base of prosternal process; the area between them extremely convex and widening anteriorly to form a chin-band, usually with coarse punctures and long pubescence (Fig. 5); body moderately large (length 2.8–5.0 mm); oblong oval, moderately convex; pronotum with the posterior angles pointed and lateral margin straight (Fig. 1). The prosternal lines of *Cryptogonus* are not as in *Aspidimerus*, varying in outline, the area enclosed by them lying at the same level as the rest of prosternum; body small, rounded oval.

#### Description.

Body moderately large, oblong oval, dorsum moderately convex, finely punctate and pubescent. Head transverse oval, eyes large, finely faceted, entire, narrowly margined and not extending to underside of head (Fig. 1). Antennae small, geniculate, composed of 8 or 9 antennomeres, antennomere 1 large, 2 slightly smaller and subtriangular, the rest together forming a spindle or an elongate oval club (Fig. 3). Terminal maxillary palpomere securiform (Fig. 4). Pronotum transverse, at middle of length twice as wide as long, strongly convex, anterior margin deeply emarginated. Scutellum subtriangular. Elytra oblong oval, moderately convex. Humeral callus rather prominent, obtusely.

Prosternum T-shaped, evenly convex (Fig. 2), prosternal lines as wide apart as the width of the base of the prosternal process, widely divergent; the area between them extremely convex and widening anteriorly to form a chin-band, usually with coarse punctures and long pubescence (Fig. 5). Both sides of prosternum deeply foveate to accommodate apices of front femora (Fig. 2). Mesoventrite transverse, widely emarginated anteriorly, indistinctly punctate and sparsely pubescent. Metaventrite usually finely punctate, with dense pubescence. Elytral epipleuron narrow, incomplete, with clearly delimited cavities to accommodate apices of mid and hind femora. Abdomen with 6 ventrites, the first being dilated posteriorly in an arc at middle, abdominal postcoxal lines incomplete (Fig. 10). Legs with femora broadly expanded, oval, and completely concealing the compressed tibiae (Figs 8–9), tarsi composed of three tarsomeres (Fig. 11).

Male genitalia: Penis curved, with a distinct penis capsule. Penis guide in ventral view flat and broad, apex pointed or truncate. Parameres slender, with sparsely distributed short setae at apex, nearly as long as penis guide. Female genitalia usually with tenth tergite broad, setose, coxites subtriangular or broad. Spermatheca vermiform, nodulus wide, ramus long.

#### Distribution.

Burma, China, India, Laos, Sri Lanka, Thailand, Vietnam.

#### Key to species of *Aspidimerus* Mulsant, 1850

**Table:** 

1	Ground color of elytra black	2
–	Ground color of elytra yellowish brown or brown	8
2	Elytra black without any spots, dorsum strongly convex and sparsely pubescent (Figs 12–14). Penis distinctly stout with pointed apex (Fig. 39), penis guide slender with hook-like apex in lateral view (Fig. 42). TL: 3.75mm, TW: 3.00mm, TH: 1.80mm. Distribution: China (Yunnan), Vietnam	*Aspidimerus nigritus* (Pang & Mao)
–	Elytra black with spots, dorsum moderately convex and densely pubescent.	3
3	Elytra black with 4 yellowish brown or red spots	4
–	Elytra black with 2 spots	5
4	Elytra black with 4 yellowish brown spots (Fig. 15). Antennae with 8 antennomeres. Penis relatively long, slender and strongly curved, apex with membranous appendage (Fig. 43). Penis guide broad, widest at basal 1/3 with truncate apex in ventral view. TL: 3.60mm, TW: 2.90mm, TH: 1.75mm. Distribution: China (Hainan, Guangxi, Taiwan)	*Aspidimerus esakii* Sasaji
–	Elytra black with 4 red spots. Antennae with 9 antennomeres. TL: 3.60–3.90 mm, TW: 2.90–3.10mm. Distribution: Vietnam	*Aspidimerus chapaensis* Hoàng
5	Penis relatively long, curved almost in a circle in whole length. Penis guide symmetrical with apex arcuate or truncate in ventral view	6
–	Penis short, curved at basal 1/3 length. Penis guide asymmetrical, with apex rounded or pointed in ventral view	7
6	Elytra black with 2 yellow spots (Fig. 18). Penis guide moderately broad, symmetrical, almost parallel-sided with arcuate apex in ventral view, distinctly shorter than parameres (Fig. 50). TL: 3.50–3.90mm, TW: 2.65–3.15mm, TH: 1.50–1.75mm. Distribution: China (Yunnan)	*Aspidimerus menglensis* Huo & Ren, sp. n.
–	Elytra black with 2 yellowish (in ♂) or reddish brown spots (in ♀). Penis guide narrow, symmetrical, slightly expanded in the middle and narrower towards the apex which is truncate. TL: 3.80–5.00mm, TW: 2.80–3.80mm. Distribution: Burma; Thailand	*Aspidimerus birmanicus* (Gorham)
7	Elytra black with 2 yellowish brown spots (Fig. 21). Penis guide broad, widest at base, gradually narrowing to the apex, apex with a lateral hook-like process in ventral view (Fig. 54). Penis guide strongly curved, widest at base, strongly narrowing to apex in lateral view (Fig. 55). TL: 2.75–2.80mm, TW: 2.25–2.30mm, TH: 1.55–1.65mm. Distribution: China (Guangxi)	*Aspidimerus guangxiensis* Yu
–	Elytra black with 2 red (sometimes yellowish brown) spots (Fig. 24). Penis guide nearly parallel-sided at basal 6/7, then strongly and asymmetrically convergent to a pointed tip in ventral view (Fig. 58). Penis guide stout, spoon-shaped in lateral view (Fig. 59). TL: 3.75–4.60mm, TW: 3.00–3.50mm, TH: 1.65–2.00mm. Distribution: China (Yunnan, Hainan, Taiwan), Vietnam	*Aspidimerus matsumurai* Sasaji
8	Ground color of elytra brown	9
–	Ground color of elytra yellowish brown	10
9	Body medium size, oblong oval. Upside clear brown with a large black marking in the middle of pronotum. TW: 4.60mm, TW: 3.40mm. Distribution: Vietnam	*Aspidimerus laokayensis* Hoàng
–	Body smaller, subrounded. Elytra brown with 4 black spots besides a black sutural spot (Fig. 27). TL: 2.85–3.50mm, TW: 2.40–2.85mm, TH: 1.25–1.75mm. Distribution: China (Guangdong, Guangxi, Yunnan), Vietnam.	*Aspidimerus kabakovi* Hoàng
10	Elytra with 8 black spots and a black sutural stripe which expanded at near basal and apical part (Fig. 30). Penis short with penis capsule extremely expanded (Fig. 65). TL: 4.10mm, TW: 3.30mm, TH: 1.46mm. Distribution: China (Yunnan)	*Aspidimerus decemmaculatus* Pang & Mao
–	Elytra with 6 black spots and a black sutural stripe which expanded near base (Figs 33, 36). Penis long with penis capsule slightly or moderately expanded	11
11	Pronotum brown; elytra without the black external border, suture with a narrow, black border between the scutellum and the middle; each elytron with 3 black spots. Distribution: India, Burma	*Aspidimerus spencii* Mulsant
–	Pronotum black; elytra with a narrow black external border all around the margins, that of the suture irregularly expanded in the basal half; each elytron with 3 spots	12
12	Penis guide stout, strongly curved in lateral view and with obtuse apex in ventral view (Figs 71–72). TL: 4.80mm, TW: 3.60mm, TH: 1.58mm. Distribution: China (Yunnan), Laos (Mouhot)	*Aspidimerus mouhoti* Crotch
–	Penis guide slender, slightly curved in lateral view and with truncate apex in ventral view	13
13	Sutural stripe irregularly expanded as Fig. 33, the elytral spot nearer the suture as large as the one situated on humeral callus (Fig. 33). Penis capsule slightly expanded (Fig. 73). Penis guide widest at basal 2/5, gradually narrowing to apex, apex truncate in ventral view (Fig. 75). TL: 4.50–4.75mm, TW: 3.40–3.60mm, TH: 2.00–2.25mm. Distribution: China (Yunnan)	*Aspidimerus zhenkangicus* Huo & Ren, sp. n.
–	Sutural stripe irregularly expanded as Fig. 36, the elytral spot nearer the suture distinctly larger than that situated on humeral callus (Fig. 36). Penis capsule moderately expanded (Fig. 79). Penis guide widest at middle, gradually narrowing to apex, margins of apical 1/6 almost parallel, apex truncate in ventral view (Fig. 81). TL: 3.75–4.00mm, TW: 3.00–3.25mm, TH: 1.55–1.65mm. Distribution: China (Sichuan, Yunnan), Vietnam, Burma	*Aspidimerus ruficrus* Gorham

#### Remarks.

*Aspidimerus nigrovittatus* Motschulsky, 1866 is not keyed in the present paper, because the description given by Motschulsky (1866) is too simple to diagnose: “Subovatus, convexus, nitidus, sparsim puberulus, pallide flavus, elytris utrinque vitta lata nigra, apice non attinguenda”. Additionally, Kapur (1948) declared that its type was not available.

### Aspidimerus nigritus

(Pang & Mao, 1979)

http://species-id.net/wiki/Aspidimerus_nigritus

Figs 12–14 , 39–42 , 85

Cryptogonus nigritus Pang & Mao, 1979: 61; Cao et al. 1992: 138.
Aspidimerus nigritus : Kovář 2007: 71, 73, 575; Ren et al. 2009: 108. Combined by Kovář 2007: 71.
Aspidimerus dongpaoensis Hoàng, 1982: 165. Synonymized by Kovář 2007: 73.

#### Diagnosis.

**Figures 12–20.**
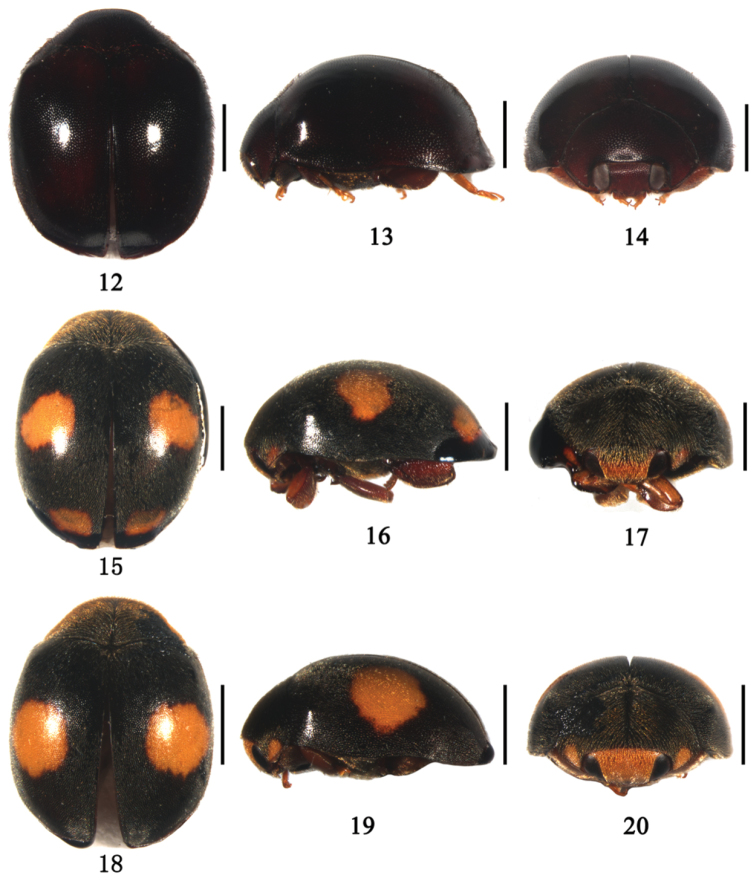
**12–14** *Aspidimerus nigritus* (Pang & Mao), **12** dorsal view **13** lateral view **14** frontal view **15–17** *Aspidimerus esakii* Sasaji, **15** dorsal view **16** lateral view **17** frontal view **18–20** *Aspidimerus menglensis* Huo & Ren, sp. n. **18** dorsal view **19** lateral view **20** frontal view. Scale bars: 1.0mm.

This is a very distinctive species with body strongly convex and dorsal surface entirely black (Figs 12–14). Penis extremely short and stout (Fig. 39). Penis guide straight with hook-like apex in lateral view (Fig. 42). In ventral view, penis guide nearly parallel at basal half and then converging gradually to a blunted tip (Fig. 41).

#### Description.

TL: 3.75mm, TW: 3.00mm, TH: 1.80mm, TL/TW: 1.25; PL/PW: 0.53; EL/EW: 0.88.

Body oblong oval, strongly convex and sparsely pubescent (Figs 12–14). Head reddish brown, eyes silver white (Fig. 14). Dorsal surface entirely black (Fig. 12). Underside reddish brown, except prosternum, mesoventrite and metaventrite dark brown.

Head small, 0.37 times elytral width (HW/EW=1:2.73). Punctures on frons sparsely distributed, separated by 1.0–3.0 times their diameter. Eyes large and almost oval, rather finely faceted, the widest interocular distance about 0.50 times head width. Punctures on dorsal surface fine, close, separated by 0.5–2.0 times their diameter, with short, thin pubescence. Pronotum 0.67 times elytral width (PW/EW=1:1.50). Surface of prosternum coarse, with sparse long pubescence. Mesoventrite with inconspicuous, rather sparse punctation. Punctures on metaventrite moderately large, separated by 0.3–1.0 times their diameter, with dense golden pubescence.

Male genitalia: Penis short, distinctly stout (Fig. 39). Penis capsule with a large outer process and an indistinct inner one (Fig. 39). Apex of penis pointed with membranous appendage (Fig. 40). Penis guide slender with hook-like apex in lateral view (Fig. 42). In ventral view, penis guide nearly parallel at basal half, and then converging gradually to a blunted tip (Fig. 41). Parameres stout, shorter than penis guide, with sparsely distributed long setae at its apex and inner side (Fig. 42).

Female genitalia: Unknown.

#### Specimens examined.

China, Yunnan: 1♂, Menglun, Jinghong, [21°55.51'N, 101°15.45'E], ca 540m, 11.v.2009, Ren SX leg (SCAU).

#### Distribution.

China (Yunnan); Vietnam.

### Aspidimerus esakii

Sasaji, 1968

http://species-id.net/wiki/Aspidimerus_esakii

Figs 15–17 , 43–47 , 85

Aspidimerus esakii Sasaji, 1968: 16; 1986: 40; Pang and Mao 1979: 54; Pang 1998: 185; Yu 1995: 139; 2011: 166; Kovář 2007: 575; Ren et al. 2009: 106.

#### Diagnosis.

This species is close to *Aspidimerus chapaensis* in general appearance, but can be separated from it by the black elytra with four yellow spots (Fig. 15), and the antennae with 8 antennomeres. In *Aspidimerus chapaensis*, elytra black with four red spots and the antennae with 9 antennomeres. The spermatheca is also diagnostic according to the illustrations given by Sasaji (1968) and Hoàng (1982).

#### Description.

TL: 3.70mm, TW: 2.90mm, TH: 1.75mm, TL/TW: 1.28; PL/PW: 0.53; EL/EW: 1.00.

Body oblong oval, dorsum moderately convex and densely pubescent (Figs 15–17). Head yellowish brown, clypeus reddish brown, eyes black (Fig. 17). Pronotum black except anterior corners yellowish brown. Scutellum and elytra black. Each elytron with two yellowish brown spots (Figs 15–16). Underside black, except legs and abdomen reddish brown.

Head small, 0.45 times elytral width (HW/EW=1:2.23). Punctures on frons small, separated by 0.5–1.0 times their diameter, with dense golden pubescence. Eyes large and oval, rather finely faceted, the widest interocular distance 0.50 times head width. Pronotum 0.69 times elytral width (PW/EW=1:1.45), punctures on pronotum and scutellum fine, close, separated by 0.5–1.0 times their diameter, with thick, golden pubescence. Elytra finely punctate, with short yellow white pubescence. Surface of prosternum coarse, with sparse, long and yellowish pubescence. Punctures on metaventrite moderately large, separated by 0.5–1.0 times their diameter, with dense yellowish pubescence.

Male genitalia: Penis relatively long, slender and strongly curved, apex of penis with membranous appendage, penis capsule with a short indistinct outer process and a long inner one (Figs 43–45). Penis guide slender, gradually tapering to apex forming a pointed tip in lateral view (Fig. 47). In ventral view, penis guide broad, widest at basal 1/3 with truncate apex (Fig. 46). Parameres slender, sparsely setose at apex, equal in length to the penis guide (Fig. 47).

#### Specimens examined.

China, Hainan: 1♂, Bawangling Natural Reserve, [19°05.49'N, 109°06.38'E], ca 260m, 5.v.2005, Wang XM leg (SCAU).

#### Distribution.

China (Hainan, Guangxi, Taiwan).

### Aspidimerus menglensis

Huo & Ren sp. n.

http://zoobank.org/6F3968D5-DEE2-40C2-84FD-8D4A0D56D175

http://species-id.net/wiki/Aspidimerus_menglensis

Figs 18–20 , 48–52 , 85

#### Diagnosis.

This species is similar to *Aspidimerus birmanicus* (Gorham) in general appearance, but can be identified by the characters as follows: penis guide moderately broad, symmetrical, almost parallel-sided with arcuate apex in ventral view, distinctly shorter than parameres (Fig. 50), while in *Aspidimerus birmanicus* (Gorham) penis guide narrow, slightly expanded in the middle and narrower towards the apex which is truncate.

It is also similar to *Aspidimerus guangxiensis* in color pattern (Figs 18, 24), but can be distinguished from the latter by its larger size and male genitalia.

**Figures 21–29.**
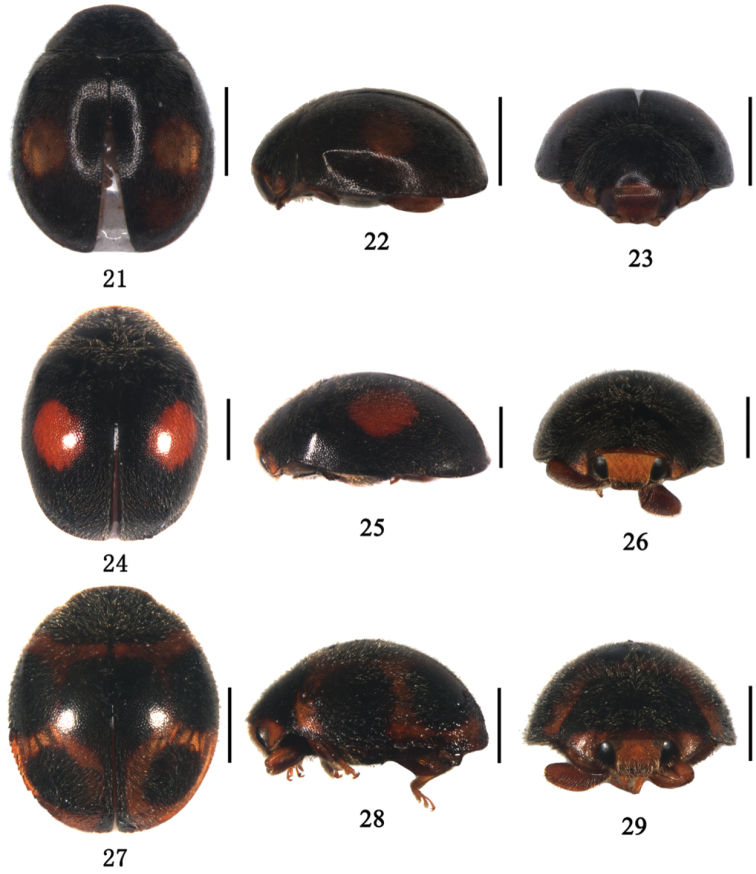
**21–23** *Aspidimerus guangxiensis* Yu, **21** dorsal view **22** lateral view **23** frontal view **24–26** *Aspidimerus matsumurai* Sasaji, **24** dorsal view **25** lateral view **26** frontal view **27–29** *Aspidimerus kabakovi* Hoàng **27** dorsal view **28** lateral view **29** frontal view. Scale bars: 1.0mm.

#### Description.

TL: 3.50–3.90mm, TW: 2.65–3.15mm, TH: 1.50–1.75mm, TL/TW: 1.24–1.32; PL/PW: 0.51–0.55; EL/EW: 1.02–1.13.

Body oval, dorsum moderately convex and pubescent (Figs 18–20). Head yellow with the base brown in male and black in female. Clypeus reddish brown. Eyes black. Pronotum black except anterior corner yellow (Fig. 20). Scutellum black. Elytra black with two round yellow spots (Fig. 18). Underside black, except mesoventrite and legs brown.

Head small, 0.47 times elytral width (HW/EW=1:2.12). Punctures on frons fine, separated by 0.2–0.5 times their diameter, with thick, golden pubescence. Eyes large and almost oval, rather finely faceted, the widest interocular distance 0.36 times head width. Pronotum 0.72 times elytral width (PW/EW=1:1.39), closely covered with small punctures and golden pubescence, denser than those on head, punctures separated by 0.5–1.0 times their diameter. Punctures on elytra very fine and close, separated by 0.5–1.5 times their diameter, with short silver white pubescence. Prosternum with sparse, coarse punctation and long pubescence. Mesoventrite small, with inconspicuous punctures and sparse pubescence. Metaventrite finely punctate, separated by 0.2–1.0 times their diameter, with pubescence dense, short and silver white.

Male genitalia: Penis long, strongly curved. Penis capsule with a short outer process and a long inner one (Fig. 48). Apex of penis with membranous appendage (Fig. 49). Penis guide slender in lateral view (Fig. 51). In ventral view, penis guide symmetrical, almost parallel-sided with arcuate apex (Fig. 50); Parameres slender, longer than penis guide, with dense long setation at its apex and inner side (Fig. 50).

Female genitalia: Tenth tergite fairly broad, 0.25 times as long as wide, with moderately long setae. Coxites subtriangular, each with a few long terminal setae (Fig. 52).

#### Types.

**Holotype**: 1♂, China, Yunnan: Mengla, Xishuangbanna, [21°26.59'N, 101°38.01'E], ca 1160 m, 29.iv.2008, Wang XM leg. (SCAU). **Paratypes** (3): Yunnan: 1♂, Nanping, Mengla, [21°39.55'N, 101°22.52'E], ca 750m, 10.v.2009, Ren SX leg. (SCAU); 1♀, Mengla, Xishuangbanna, [21°26.59'N, 101°38.01'E], ca 1160m, 29.iv.2008, Wang XM leg. (SCAU); 1♂, Longmen, Mengla, [21°30.17'N, 101°31.44'E], ca 760m, 1.v.2008, Wang XM leg. (SCAU).

#### Distribution.

China (Yunnan).

#### Etymology.

The specific epithet refers to the type locality, Mengla, Yunnan.

### Aspidimerus guangxiensis

Yu, 2004

http://species-id.net/wiki/Aspidimerus_guangxiensis

Figs 21–23 , 53–55 , 85

Aspidimerus guangxiensis Yu, 2004: 329; Ren et al. 2009: 106.

#### Diagnosis.

This species is similar to *Aspidimerus matsumurai* Sasajiin elytra with two spots (Figs 21, 24), but can be distinguished from the latter by its smaller size and unique male genitalia: penis short, apical 1/2 length with membranous appendage (Fig. 53), penis guide extremely broad, widest at base, gradually narrowing to the apex, apex with a lateral, hook-like process in ventral view (Fig. 54). In *Aspidimerus matsumurai*,penis short, apical 2/5 length with less membranous appendage (Fig. 56), penis guide broad in ventral view, nearly parallel-sided at basal 6/7, then strongly and asymmetrically convergent to a pointed tip (Fig. 58).

#### Description.

TL: 2.75–2.80mm, TW: 2.25–2.30mm, TH: 1.55–1.65mm, TL/TW: 1.25–1.30; PL/PW: 0.50–0.55; EL/EW: 0.91–1.11.

Body small, oval, moderately convex, dorsal surface pubescent (Figs 21–23). Head yellow or reddish brown with eyes black (Fig. 23). Pronotum black, with basal margin reddish brown and anterior corners yellowish brown. Scutellum and elytra black. Each elytron with one yellowish brown spot, rounded, situated at the middle of elytron (Fig. 21). Underside dark brown, except legs and abdomen reddish brown.

Head small, 0.44 times elytral width (HW/EW=1:2.25). Punctures on frons fine, separated by 1.0–2.0 times their diameter, with short silver white setae. Eyes small, broadly oval, widest interocular distance 0.55 times head width (Fig. 20). Pronotum 0.67 times elytral width (PW/EW=1:1.50). Pronotal punctures fine, separated by 1.0–3.0 times their diameter. Scutellum triangular. Punctures on elytra slightly larger than those on pronotum, separated by 1.0–2.0 times their diameter. Prosternum coarse, with sparse punctation and long pubescence. Mesoventrite small, with a few setae. Metaventrite densely pubescent with punctures large and closely spaced, separated by 0.5–1.0 times their diameter.

Male genitalia: Penis short and stout. Penis capsule with a distinctly outer process and a long inner one (Fig. 53). Penis guide widest at base and abruptly constricted forming a pointed apex in lateral view (Fig. 55). In ventral view, penis guide flat and asymmetrical, widest at base, gradually narrowing to the apex, apex with a lateral, hook-like process (Fig. 54). Parameres slender, sparsely setose at apex, shorter than penis guide (Fig. 55).

Female genitalia: Unknown.

#### Specimens examined.

China, Guangxi: 1♂, Shiwandashan Natural Reserve, Shangsi, [21°54.36'N, 107°54.28'E], ca 300m, 27.vii.2005, Zhang CW leg(SCAU); 1♂, Fulong, Fangchenggang, [22°51.03'N, 107°55.43'E], ca 230m, 28.vii.2005, Wang XM leg (SCAU).

#### Distribution.

China (Guangxi).

### Aspidimerus matsumurai

Sasaji, 1968

http://species-id.net/wiki/Aspidimerus_matsumurai

Figs 24–26 , 56–60 , 85

Aspidimerus matsumurai Sasaji, 1968: 17; Pang and Mao 1979: 53; Hoàng 1982: 162; Cao and Xiao 1984: 98; Cao et al. 1992: 131; Pang 1998: 185; Kovář 2007: 575; Ren et al. 2009: 108; Yu 2011: 167.

#### Diagnosis.

This species is close to *Aspidimerus guangxiensis* and *Aspidimerus birmanicus* (Gorham) in dorsal coloration, but can be identified by the following characters: Penis stout and short. Penis capsule with a small inner process (Fig. 56). In ventral view, penis guide extremely broad, nearly parallel-sided at basal 6/7, then strongly and asymmetrically convergent to a pointed tip (Fig. 58). In *Aspidimerus guangxiensis*, penis guide widest at base, gradually narrowing to the apex, apex with a lateral hook-like process in ventral view (Fig. 54). In *Aspidimerus birmanicus*, penis relatively long, penis capsule with a long inner process, penis guide distinctly narrower than that of *Aspidimerus matsumurai* in ventral view.

*Aspidimerus matsumurai* is also similar to *Aspidimerus laokayensis* in male genitalia, but can be distinguished from the latter by its color pattern and detailed structure of genitalia.

#### Description.

TL: 3.75–4.60mm, TW: 3.00–3.50mm, TH: 1.65–2.00mm, TL/TW: 1.25–1.31; PL/PW: 0.50–0.51; EL/EW: 0.97–1.03.

Body oblong oval, moderately convex and pubescent (Figs 24–26). Head yellow with clypeus dark brown and eyes black (Fig. 26). Pronotum black except antero-lateral corners and anterior margin yellowish brown (Fig. 26). Scutellum black. Elytra black with two round red (sometimes yellowish brown) spots (Fig. 24). Underside black, except legs and abdomen reddish brown.

Head small, 0.42 times elytral width (HW/EW=1:2.40). Punctures on frons close, separated by 0.3–0.5 times their diameter, with thin, yellow white pubescence. Eyes large and broadly oval, rather finely faceted, widest interocular distance 0.60 times head width, posterior of the eye with silver setae. Pronotum 0.73 times elytral width (PW/EW=1:1.36). Pronotal punctures similar to those on head, separated by 0.5–1.5 times their diameter, with thick silver white pubescence. Punctures on elytra slightly larger than those on pronotum, separated by 0.5–1.0 times their diameter. Prosternum coarsely punctate, with pubescence sparse, long and yellowish. Mesoventrite indistinctly punctate, with several hairs. Punctures on metaventrite fine, separated by 0.2–0.5 times their diameter, with long setae.

Male genitalia: Penis stout and short, curved at basal 1/3, gradually narrowing to the apex (Fig. 56). Apical 2/5 length of penis with membranous appendage. Penis capsule with a large outer process and a small inner one (Figs 56–57). Penis guide stout, spoon-shaped in lateral view (Fig. 59). In ventral view, penis guide very broad, nearly parallel-sided, slightly divergent apically, apical part asymmetrical, suddenly and strongly convergent to a blunted tip (Fig. 58). Parameres slender, sparsely setose at apex, slightly shorter than penis guide (Fig. 59).

Female genitalia: Tenth tergite broad, arc-shaped with terminal setae. Coxites broad, 0.4 times as long as wide, each with moderately long terminal setae (Fig. 60); spermatheca absent.

#### Specimens examined.

China, Yunnan: 1 male, Nanping, Mengla, [21°39.55'N, 101°22.52'E], ca 740m, 16.v.2008, Ren SX leg (SCAU); Hainan: 1♂ 1♀, Diaoluoshan National Forest Park, [18°47.30'N, 109°52.58'E], ca 280m, 8.v.2005, Wang XM leg (SCAU).

#### Distribution.

China (Yunnan, Hainan, Taiwan); Vietnam.

### Aspidimerus kabakovi

Hoàng, 1982

http://species-id.net/wiki/Aspidimerus_kabakovi

Figs 27–29 , 61–64 , 86

Aspidimerus kabakovi Hoàng, 1982: 167.

#### Diagnosis.

This species can be easily distinguished by the following characters: elytra brown with 5 subrounded black spots disposed as Fig. 27. Apical 1/3 length of penis is very characteristic (Fig. 61). Penis guide broad, basal 2/3 length nearly parallel-sided, apical 1/3 strongly convergent with rounded apex (Fig. 62).

#### Description.

TL: 2.85–3.50mm, TW: 2.40–2.85mm, TH: 1.25–1.75mm, TL/TW: 1.19–1.23; PL/PW: 0.52; EL/EW: 0.94–0.96.

Body subrounded, dorsum moderately convex and pubescent (Figs 27–29). Head yellowish brown with clypeus reddish brown, eyes black or silver white. Pronotum black except anterior margin and anterior corners brown (Fig. 29). Scutellum black. Elytra brown with 5 subrounded black spots arranged as follows: 1 on the middle of suture and 2 on each elytron, the front larger, confluent with the border (Figs 27–28). Underside reddish brown, except prosternum, mesoventrite and metaventrite dark brown.

Head small, 0.42 times elytral width (HW/EW=1:2.40). Punctures on frons small, separated by 0.5–1.0 times their diameter. Eyes large, widest interocular distance 0.60 times head width. Dorsal surface finely punctate, with dense, silver white pubescence. Pronotum 0.69 times elytral width (PW/EW=1:1.45). Underside finely punctate, with pubescence dense, short and silver white.

Male genitalia: Penis stout with apical 1/3 length very characteristic as shown in Fig. 61. Penis capsule with distinct outer and inner processes (Fig. 61). Penis guide stout and slightly curved in lateral view (Fig. 63). In ventral view, penis guide broad, basal 2/3 length nearly parallel-sided, apical 1/3 strongly convergent with rounded apex (Fig. 62). Parameres slender, as long as the penis guide, sparsely setose at apex (Fig. 62).

Female genitalia: tenth tergite fairly broad, 0.15 times as long as wide, setaceous at apex, coxites subtriangular with a few long terminal setae (Fig. 64); spermatheca absent.

#### Specimens examined.

China, Guangdong: 1♂, Nankunshan Natural Reserve, Huizhou, [23°37.54'N, 113°52.56'E], ca 490m, 5.viii.1986, Pang XF leg (SCAU); Guangxi: 1♀, Shiwandashan Natural Reserve, Shangsi, [21°54.25'N, 107°54.33'E], ca 380m, 9.ix.2004, Lv XB leg (SCAU); 1♂, Maoershan Natural Reserve, 18.x.2004, [25°51.50'N, 110°25.10'E], ca 1350m,Wang XM leg (SCAU); 1♂, Fulong, Fangchenggang, [22°05.19'N, 107°59.28'E], ca 220m, 29.vii.2005, Qin ZQ leg (SCAU); Yunnan: 1♀, Dajianshan, Pingbian, [22°54.14'N, 103°41.52'E], 2100m, 20.iv.2008, Wang XM leg (SCAU).

#### Distribution.

China (Guangdong, Guangxi, Yunnan); Vietnam.

### Aspidimerus decemmaculatus

Pang & Mao, 1979

http://species-id.net/wiki/Aspidimerus_decemmaculatus

**Figures 30–38.**
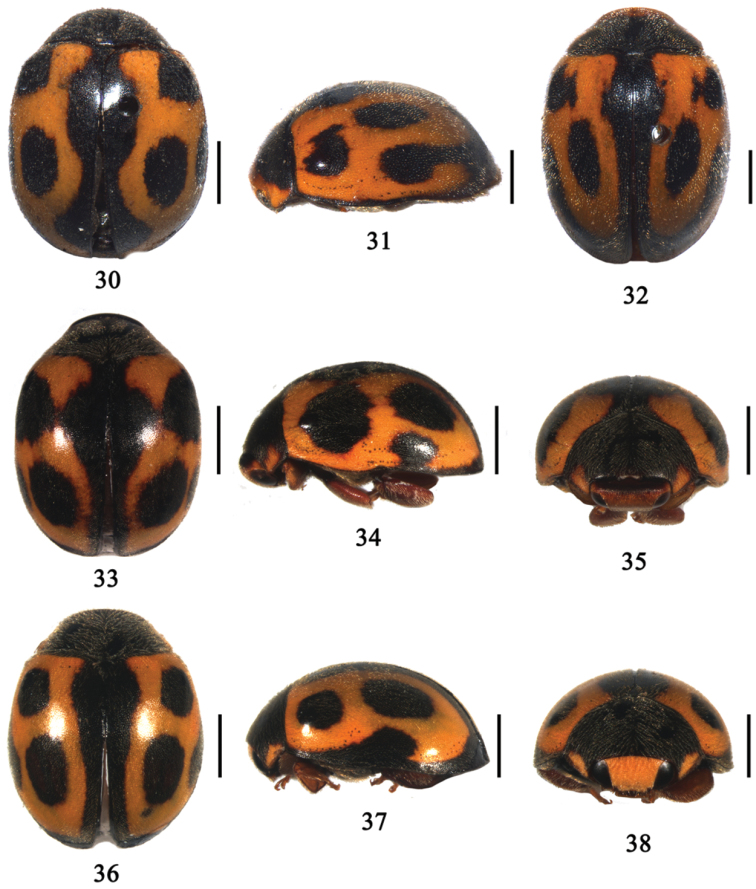
**30** *Aspidimerus decemmaculatus* Pang & Mao, dorsal view; **31–32** *Aspidimerus mouhoti* Crotch, **31** lateral view **32** dorsal view **33–35** *Aspidimerus zhenkangicus* Huo & Ren, sp. n. **33** dorsal view **34** lateral view **35** frontal view **36–38** *Aspidimerus ruficrus* Gorham, **36** dorsal view **37** lateral view **38** frontal view. Scale bars: 1.0mm.

Figs 30 , 65–68 , 86

Aspidimerus decemmaculatus Pang & Mao, 1979: 56; Cao and Xiao 1984: 98; Cao et al. 1992: 134; Pang 1998: 186; Kovář 2007: 575; Ren et al. 2009: 106.

#### Diagnosis.

This species can be easily distinguished from other *Aspidimerus* bythe following characters: elytra with 8 black spots and a black sutural stripe which expanded at near basal and apical part (Fig. 30). Penis capsule extremely expanded (Fig. 65). Penis guide subtriangular, widest at base with rounded apex in ventral view (Fig. 67).

**Figures 39–52.**
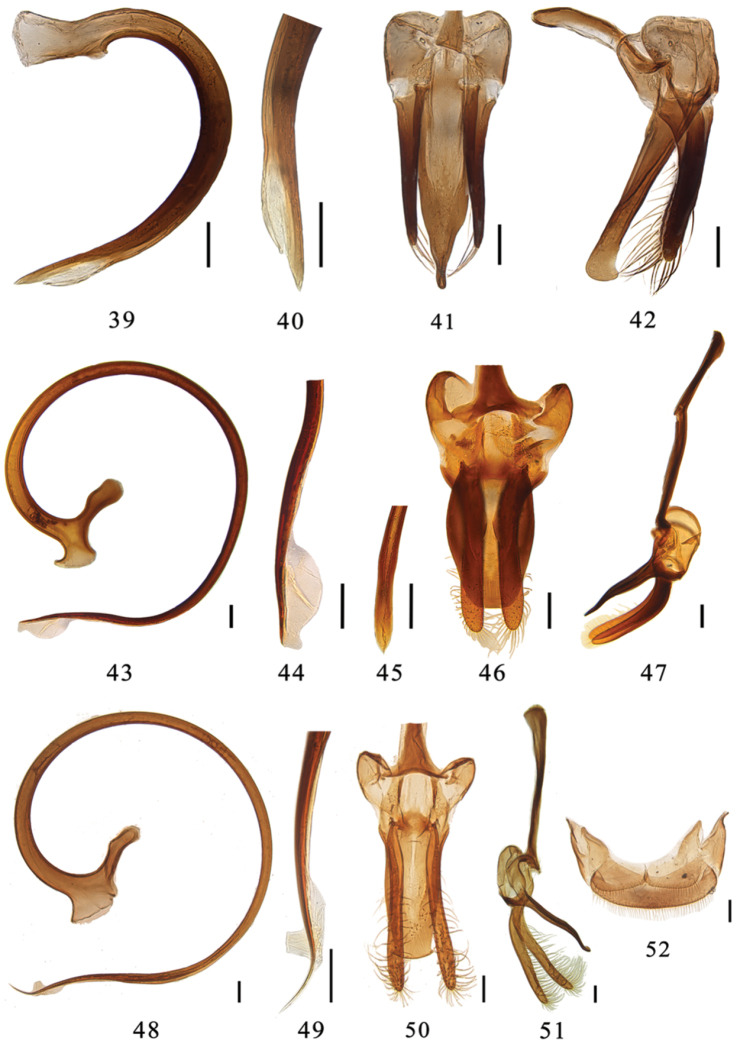
**39–42** *Aspidimerus nigritus* (Pang & Mao), male genitalia: **39** penis **40** apex of penis **41** tegmen, ventral view **42** tegmen, lateral view **43–47** *Aspidimerus esakii* Sasaji, male genitalia: **43** penis **44** apex of penis **45** apex of penis, ventral view **46** tegmen, ventral view **47** tegmen, lateral view **48–52** *Aspidimerus menglensis* Huo & Ren, sp. n. **48–51** male genitalia: **48** penis **49** apex of penis **50** tegmen, ventral view **51** tegmen, lateral view **52** female genitalia: ovipositor. Scale bars: 0.1mm.

**Figures 53–64.**
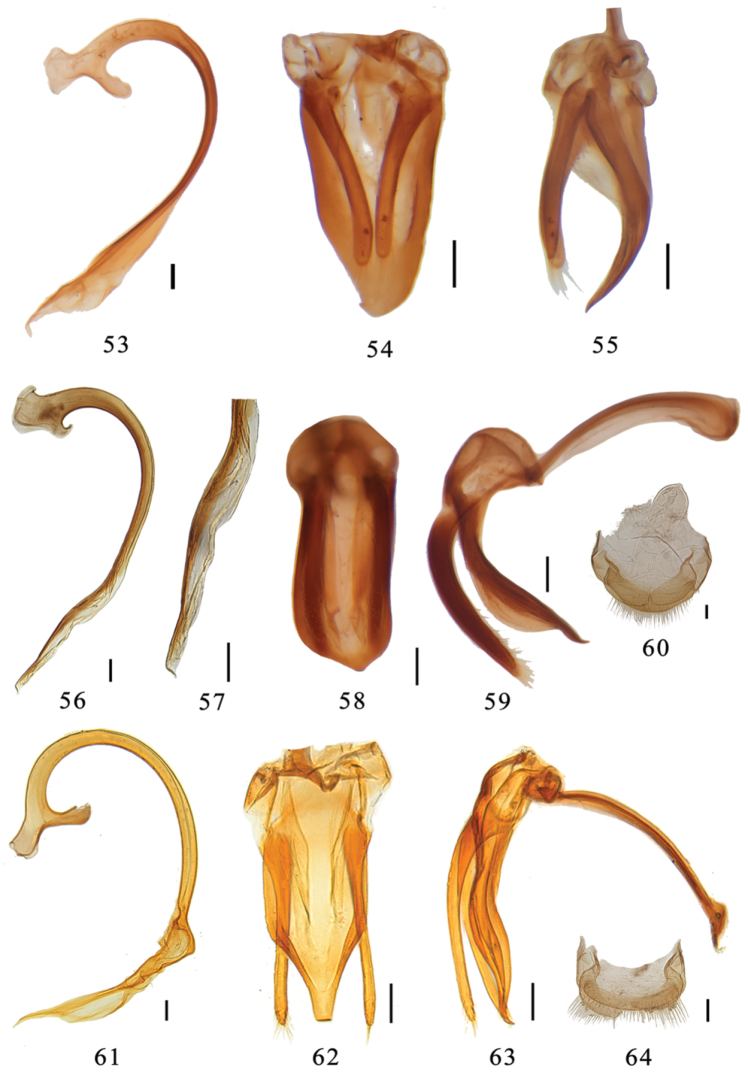
**53–55** *Aspidimerus guangxiensis* Yu, male genitalia: **53** penis **54** tegmen, ventral view **55** tegmen, lateral view **56–60** *Aspidimerus matsumurai* Sasaji **56–59** male genitalia: **56** penis **57** apex of penis **58** tegmen, ventral view **59** tegmen, lateral view **60** female genitalia: ovipositor **61–64** *Aspidimerus kabakovi* Hoàng **61–63** male genitalia **61** penis **62** tegmen, ventral view **63** tegmen, lateral view **64** female genitalia: ovipositor. Scale bars: 0.1mm.

**Figures 65–78.**
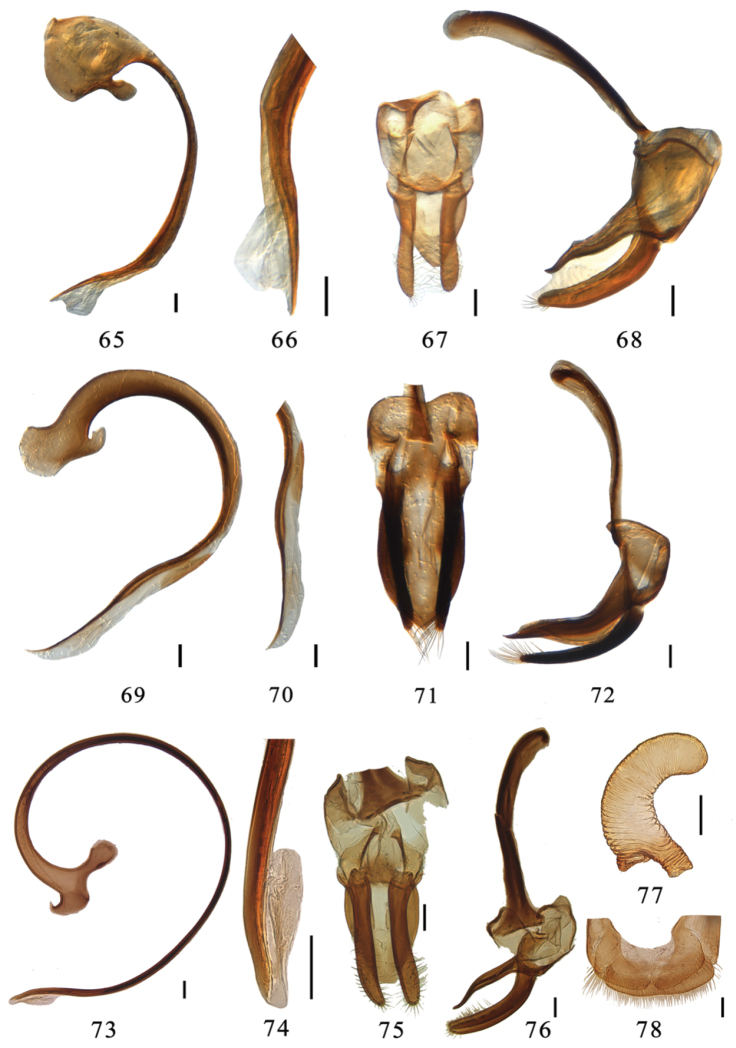
**65–68** *Aspidimerus decemmaculatus* Pang & Mao, male genitalia: **65** penis **66** apex of penis **67** tegmen, ventral view **68** tegmen, lateral view **69–72** *Aspidimerus mouhoti* Crotch, male genitalia: **69** penis **70** apex of penis **71** tegmen, ventral view **72** tegmen, lateral view **73–78** *Aspidimerus zhenkangicus* Huo & Ren, sp. n.**73–76** male genitalia: **73** penis **74** apex of penis **75** tegmen, ventral view **76** tegmen, lateral view **77–78** female genitalia: **77** spermatheca **78** ovipositor. Scale bars: 0.1mm.

#### Description.

TL: 4.10mm, TW: 3.30mm, TH: 1.46mm, TL/TW: 1.24; PL/PW: 0.39; EL/EW: 1.09.

Body large, oblong oval, dorsum moderately convex and pubescent (Fig. 30). Head yellowish brown with eyes black. Pronotum black, with anterior corners yellowish brown. Scutellum black. Elytra yellowish brown. Elytral margins black. Each elytron with 4 black spots besides a black sutural stripe which expanded at near basal and apical part. Elytral spots arranged as follows: spot 1 triangle, situated on humeral callus, spot 2 subrounded, posterior to the transverse middle line, nearer the suture, spot 3 small, oblong, and confluent with the border, spot 4 small, prior apex, confluent with the border (Fig. 30). Underside black, except legs and abdomen reddish brown.

Head small, 0.38 times elytral width (HW/EW=1:2.64). Punctures on frons fine, separated by 0.5–1.0 times their diameter, with thin, yellow white pubescence. Eyes large and almost oval, finely faceted, the widest interocular distance 0.56 times head width. Pronotum 0.60 times elytral width (PW/EW=1:1.67). Punctures on pronotum and elytra close, separated by 0.5–1.0 times their diameter, with thick, yellowish pubescence. Underside coarsely punctate, with sparse yellowish pubescence.

Male genitalia: Penis short, penis capsule with an extremely expanded outer process and a short inner process (Fig. 65). Apex of penis with membranous appendage (Fig. 66). In ventral view, penis guide subtriangular, widest at base with a rounded apex (Fig. 67). Penis guide slender and waved, gradually tapering to apex in lateral view, parameres slender, sparsely setose at apex, slightly shorter than penis guide (Fig. 68).

Female genitalia: Unknown.

#### Specimens examined.

**Holotype**: 1♂, China, Yunnan: Mengzhe, Xishuangbanna, [22°01.42'N, 100°17.41'E], ca 1350m, 26.vi.1958, Wang SY leg. (IOZ).

#### Distribution.

China (Yunnan).

### Aspidimerus mouhoti

Crotch, 1874

http://species-id.net/wiki/Aspidimerus_mouhoti

Figs 31–32 , 69–72 , 86

Aspidimerus mouhoti Crotch, 1874: 202; Korschefsky 1931: 172.
Aspidimerus sexmaculatus Pang & Mao, 1979: 55; Cao and Xiao 1984: 98; Cao et al. 1992: 133; Pang 1998: 186; Kovář 2007: 73, 575; Ren et al. 2009: 110. Synonymized by Kovář 2007: 73.

#### Diagnosis.

This species is similar to *Aspidimerus ruficrus* in general appearance, but can be distinguished by the stout, strongly curved penis guide in lateral view and symmetrical with obtuse apex in ventral view (Figs 71–72). In *Aspidimerus ruficrus*, penis guide slender, slightly straight in lateral view and vase-shaped with truncate apex in ventral view (Figs 81–82). The shape of penis is also diagnostic (Fig. 69).

**Figures 79–84.**
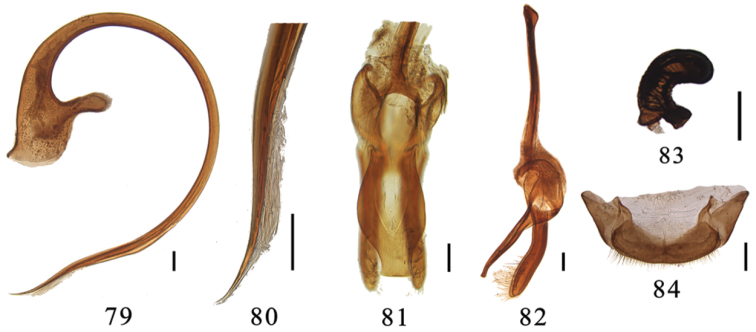
*Aspidimerus ruficrus* Gorham, **79–82** male genitalia: **79** penis **80** apex of penis **81** tegmen, ventral view **82** tegmen, lateral view **83–84** female genitalia: **83** spermatheca **84** ovipositor. Scale bars: 0.1mm.

#### Description.

TL: 4.80mm, TW: 3.60mm, TH: 1.58mm, TL/TW: 1.33; PL/PW: 0.33; EL/EW: 1.14.

Body relatively large, oval, dorsum convex and pubescent (Fig. 32). Head yellowish brown in male and reddish brown in female. Eyes black. Clypeus, mouthparts and antennae reddish brown. Pronotum black with anterior corner yellowish brown (Fig. 31). Scutellum black. Elytra yellowish brown with 3 black spots on each elytron, spot 1 subrounded, situated on humeral callus, spot 2 long oval, largest, situated on middle of elytra, spot 3 smaller, oblong, and extending to the apex confluent with the border (Figs 31–32). Lateral margins black. Underside reddish brown except prosternum, mesoventrite and metaventrite black.

Head transverse and ventrally flattened, 0.36 times elytral width (HW/EW=1:2.77). Punctures on frons moderately large, separated by 0.5–1.0 times their diameter, with short sparsely distributed setae. Eyes moderately large and finely faceted, widest interocular distance 0.62 times head width. Pronotum 0.70 times elytral width (PW/EW=1:1.43), closely covered with fine punctures associated with long dense pubescence, punctures smaller than those on head, separated by 0.5–1.5 times their diameter. Punctures on elytra very fine and close, similar to those on pronotum, with dense silver white pubescence. Prosternum coarse, with sparse long yellowish pubescence. Punctures on metaventrite moderately large, separated by 0.5–1.0 times their diameter, with dense yellowish pubescence.

Male genitalia: Penis short and stout, strongly curved at basal half, apical half with membranous appendage (Figs 69–70). Penis capsule with a large outer process and a small, unciform inner process (Fig. 69). Penis guide stout and strongly curved in lateral view (Fig. 72). In ventral view, penis guide symmetrical, widest at middle and converging gradually to a blunted tip (Fig. 71). Parameres slender, equal in length to penis guide, sparsely setose at apex (Fig. 72)

Female genitalia: Unknown.

#### Specimens examined.

1♂, China, Yunnan: Menghun, Xishuangbanna, [21°50.56'N, 100°23.02'E], ca 1200m, 15.vi.1958, Pu FJ leg. (Holotype of *Aspidimerussexmaculatus* Pang & Mao, IOZ).

#### Distribution.

China (Yunnan); Laos (Mouhot).

**Figures 85.**
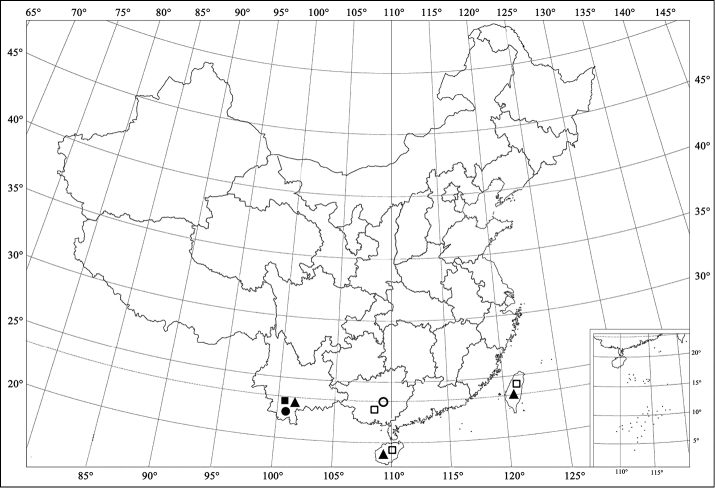
Distribution map. *Aspidimerusnigritus* (Pang & Mao) (*■*); *Aspidimerus esakii* Sasaji (*□*); *Aspidimerus menglensis* Huo & Ren, sp. n. (*●*); *Aspidimerusguangxiensis* Yu (*○*); *Aspidimerus matsumurai* Sasaji (*▲*).

**Figures 86.**
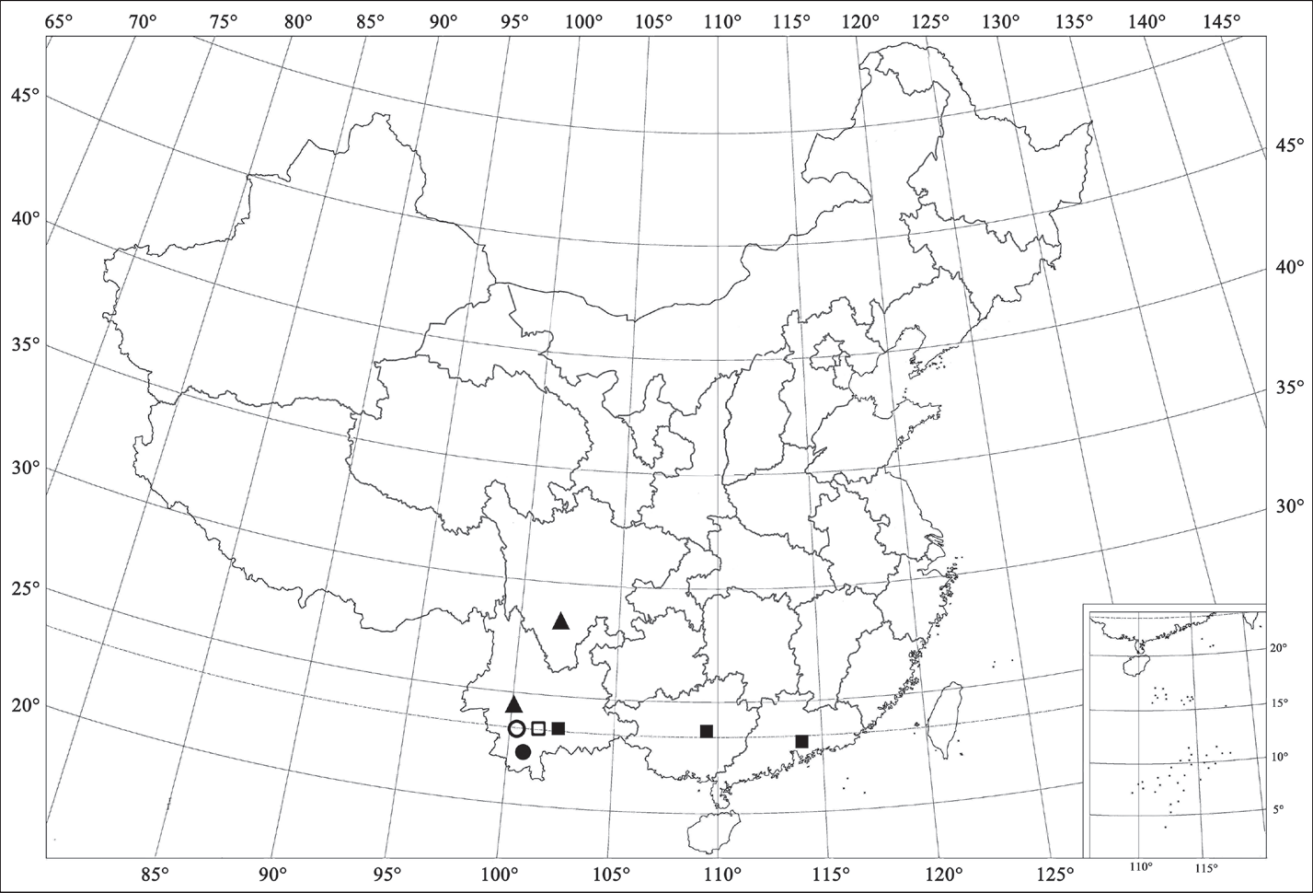
Distribution map. *Aspidimerus kabakovi* Hoàng (*■*); *Aspidimerus decemmaculatus* Pang & Mao (*□*); *Aspidimerusmouhoti* Crotch (*●*); *Aspidimerus zhenkangicus* Huo & Ren, sp. n. (*○*); *Aspidimerusruficrus* Gorham (*▲*).

### Aspidimerus zhenkangicus

Huo & Ren sp. n.

http://zoobank.org/F3B523AB-4599-4496-BD86-D437E3C60D1E

http://species-id.net/wiki/Aspidimerus_zhenkangicus

Figs 33–35 , 73–78 , 86

#### Diagnosis.

This species closely resembles *Aspidimerus ruficrus* in elytral color pattern (Figs 33, 36), but male genitalia are quite different (Figs 73–76; 79–82). It is also similar to *Aspidimerus esakii* in male genitalia, but can be distinguished from the latter by the elytra yellow with 7 black spots (Fig. 33), apex of penis with relatively small membranous appendage (Fig. 73), different shape of penis capsule (Figs 43, 73) and more narrowly truncate apex of penis guide in ventral view (Figs 46, 75). The spermatheca (Fig. 77) is also different from that of *Aspidimerus esakii* given by Sasaji (1968).

#### Description.

TL: 4.50–4.75mm, TW: 3.40–3.60mm, TH: 2.00–2.25mm, TL/TW: 1.32; PL/PW: 0.54–0.56; EL/EW: 1.04–1.06.

Body medium size and oval, dorsum moderately convex and pubescent (Fig. 33–35). Head dark yellow with basal margin and clypeus reddish brown, eyes black. Pronotum black, except anterior corners dark yellow (Fig. 35). Scutellum black. Elytra yellowish brown with black sutural stripe distinctly expanded near basal half (Fig. 33). Elytral margins black. Each elytron with 3 black spots arranged as Fig. 34. Underside reddish brown, except legs dark yellow.

Head small, 0.44 times elytral width (HW/EW=1:2.27). Surface of head with fine punctures, separated by 0.3–1.0 times their diameter, with thick, yellowish pubescence. Eyes large and almost oval, rather finely faceted, the widest interocular distance 0.50 times head width. Pronotum 0.71 times elytral width (PW/EW=1:1.42). Punctation on pronotum sparser than on head, separated by 1.0–1.5 times their diameter, with thick, yellowish pubescence. Surface of elytra densely pubescent and sparsely punctate, punctures separated by 0.5–2.0 times their diameter. Underside finely punctate, with pubescence dense, moderately long and yellowish.

Male genitalia: Penis relatively long, slender, curved almost in a circle. Penis capsule with a short outer process and a long inner one, apex of penis with relatively small membranous appendage (Figs 73–74). Penis guide slender, gradually tapering to apex in lateral view (Fig. 76). In ventral view, penis guide short oval, widest at base with truncate apex (Fig. 75). Parameres slender, densely setose along almost half of their length, longer than penis guide (Fig. 76).

Female genitalia: Tenth tergite broad with terminal setae. Coxites subtriangular with moderately long terminal setae (Fig. 78). Spermatheca vermiform with wide nodulus and long ramus (Fig. 77).

#### Types.

**Holotype**: 1♂, China, Yunnan: Mengdui, Zhenkang, [23°53.47'N, 98°53.33'E], ca 1400m, 18.vi.2008, Wang XM leg. (SCAU). **Paratypes** (5): Yunnan: 1♂, Longmen, Mengla, [21°15.12'N, 101°38.52'E], ca 1030m, 9.v.2009, Ren SX leg. (SCAU); 1♂, Yaoqu, Mengla, [21°41.22'N, 101°34.07'E], ca 700m, 7.v.2009, Ren SX leg. (SCAU); 1♂ 1♀, Dadugang, Jinghong, [22°21.39'N, 100°54.28'E], ca 1050m, 5.v.2009, Wang XM leg. (SCAU); 1♀, Xiaomengyang, Xishuangbanna, [22°04.50'N, 100°54.18'E], ca 790m, 27.iv.2008, Wang XM leg. (SCAU).

#### Distribution.

China (Yunnan).

#### Etymology.

The specific epithet refers to the location of the holotype, Zhenkang, Yunnan.

### Aspidimerus ruficrus

Gorham, 1895

http://species-id.net/wiki/Aspidimerus_ruficrus

Figs 36–38 , 79–84 , 86

Aspidimerus ruficrus Gorham, 1895: 690; Korschefsky 1931: 173; Kapur 1948: 83; Pang and Mao 1979: 55; Cao and Xiao 1984: 98; Cao et al. 1992: 134; Pang 1998: 186; Kovář 2007: 575; Ren et al. 2009: 108; Poorani 2002: 343.
Cryptogonus blandus Mader, 1954: 130; Liu 1963: 84. **syn. n.**
As pidimerus blandus (Mader, 1954): Kovář 2007: 73. Combined by Kovář 2007: 73.

#### Diagnosis.

This species is similar to *Aspidimerus mouhoti* in general appearance, but can be distinguished by the penis guide slender and slightly straight in lateral view and vase-shaped with truncate apex in ventral view (Figs 81–82). The shape of penis is also diagnostic (Fig. 79).

#### Description.

TL: 3.75–4.00mm, TW: 3.00–3.25mm, TH: 1.55–1.65mm, TL/TW: 1.23–1.25; PL/PW: 0.54–0.56; EL/EW: 0.98–1.00.

Body oblong oval, dorsum moderately convex and pubescent (Figs 36–38). Head deep yellow in male and black in female. Eyes black or silver. Pronotum black with anterior corners deep yellow (Fig. 38). Scutellum black. Elytra deep yellow with a black border along all the margins and three black spots (Figs 36–37). Spot 1 rounded, situated on humeral callus. Spot 2 subrounded, largest, posterior to the transverse middle line, nearer the suture. Spot 3 small, oblong, and confluent with the lateral margin. Underside black, except legs and abdomen reddish brown.

Head small and transverse, 0.43 times elytral width (HW/EW=1:2.31). Punctures on frons finer, separated by 0.5–1.0 times their diameter, with thin, yellow white pubescence. Eyes moderately large and rather finely faceted, the widest interocular distance 0.31 times head width. Pronotum convex and transverse, 0.68 times elytral width (PW/EW=1:1.46), covered with finely close punctures associated with dense yellowish pubescence. Punctures on elytra fine and close, smaller than those on pronotum, separated by 1.0–2.0 times their diameter, with dense silver white pubescence. Scutellum subtriangular. Punctures on prosternum coarse, with sparse long pubescence. Mesoventrite with yellow white pubescence. Metaventrite coarsely punctate, separated by 0.3–0.5 times their diameter, with pubescence sparse, moderately long and yellowish.

Male genitalia: Penis long, curved almost in a circle. Penis capsule with an expanded outer process and a small inner one (Fig. 79). Apex of penis with membranous appendage (Fig. 80). Penis guide slender and nearly straight in lateral view (Fig. 82). In ventral view, penis guide vase-shaped, widest at middle, with truncate apex (Fig. 81). Parameres slender, equal in length to penis guide, sparsely setose at apex (Fig. 82).

Female genitalia: Tenth tergite moderately broad and coxites subtriangular, each with a few long terminal setae (Fig. 84). Spermatheca with a wide nodulus, a stout cornu and a short ramus (Fig. 83).

#### Specimens examined.

**China**, **Yunnan**: 2♂ 1♀, Longmen, Mengla, [21°15.12'N, 101°38.52'E], ca 1030m, 9.v.2009, Ren SX leg (SCAU); 1♀, Longmen, Mengla, [21°30.17'N, 101°31.44'E], ca 760m, 1.v.2008, Wang XM leg (SCAU); 1♀, Mengdui, Zhenkang, [23°53.47'N, 98°53.33'E], 1400m, 18.v.2008, Wang XM leg (SCAU).

#### Distribution.

China (Sichuan, Yunnan); Vietnam; Burma.

#### Remarks.

Mader (1954) described the species *Cryptogonusblandus* based on 1 male and 3 female specimens which were collected from Yunnan, China. Liu (1963) recorded *Cryptogonus blandus* Mader in his monography and mentioned that this species belongs to the genus *Aspidimerus* according to the character of its prosternal lines. Kovář (2007) transferred *Cryptogonus blandus* to the genus *Aspidimerus* without further explanation. An examination of the specimens of *Aspidimerus blandus* (Mader) collected from type locality show that the characters of the adult, including the male genitalia, were in perfect agreement with the descriptions and illustrations of *Aspidimerus ruficrus* given by Kapur (1948). Therefore, we considered *Aspidimerus blandus* (Mader, 1954) as a junior synonym of *Aspidimerus ruficrus* Gorham, 1895.

### Catalogue of *Aspidimerus* Mulsant, 1850

*Aspidimerus* Mulsant, 1850: 944. Type species: *Aspidimerusspencii* Mulsant, 1850.

*Aspidimerus* Mulsant: Gorham 1895: 690. Crotch 1874: 202; Weise 1885: 232; 1900: 426; Mader 1926: 16; Korschefsky 1931: 172; Kapur 1948: 81; Sasaji 1968: 15; Pang and Mao 1979: 53; Hoàng 1982: 161; Pang 1998: 185; Poorani 2002: 343; Yu and Li 2004: 329; Ren et al. 2009: 106.

***Aspidimerus birmanicus*** (Gorham, 1895)

*Cryptogonus birmanicus* Gorham, 1895: 691.

*Aspidimerus birmanicus* (Gorham): Kapur 1948: 84; Poorani 2002: 343. Combined by Kapur 1948: 84.

**Distribution.** Burma; Thailand.

***Aspidimerus chapaensis*** Hoàng, 1982

*Aspidimerus chapaensis* Hoàng, 1982: 166.

**Distribution.** Vietnam.

***Aspidimerus decemmaculatus*** Pang & Mao, 1979

*Aspidimerus decemmaculatus* Pang & Mao, 1979: 56; Cao and Xiao 1984: 98; Cao et al. 1992: 134; Pang 1998: 186; Kovář 2007: 575; Ren et al. 2009: 106.

**Distribution.** China (Yunnan).

***Aspidimerus esakii*** Sasaji, 1968

*Aspidimerus esakii* Sasaji, 1968: 16; Pang and Mao 1979: 54; Pang 1998: 185; Yu 1995: 139; 2011: 166; Kovář 2007: 575; Ren et al. 2009: 106.

**Distribution.** China (Hainan, Guangxi, Taiwan).

***Aspidimerus guangxiensis*** Yu, 2004

*Aspidimerus guangxiensis* Yu, 2004: 329; Ren et al. 2009: 106.

**Distribution.** China (Guangxi).

***Aspidimerus kabakovi*** Hoàng, 1982

*Aspidimerus kabakovi* Hoàng, 1982: 167.

**Distribution.** China (Guangdong, Guangxi, Yunnan); Vietnam.

***Aspidimerus laokayensis*** Hoàng, 1982

*Aspidimerus laokayensis* Hoàng, 1982: 164.

**Distribution.** Vietnam.

***Aspidimerus matsumurai*** Sasaji, 1968

*Aspidimerus matsumurai* Sasaji, 1968: 17; Pang and Mao 1979: 53; Hoàng 1982: 162; Cao and Xiao 1984: 98; Cao et al. 1992: 131; Pang 1998: 185; Kovář 2007: 575; Ren et al. 2009: 108; Yu 2011: 167.

**Distribution.** China (Yunnan, Hainan, Taiwan); Vietnam.

***Aspidimerus menglensis*** Huo & Ren, sp. n.

*Aspidimerus menglensis* Huo & Ren, sp. n.

**Distribution.** China (Yunnan).

***Aspidimerus mouhoti*** Crotch, 1874

*Aspidimerus mouhoti* Crotch, 1874: 202. Korschefsky 1931: 172.

*Aspidimerus sexmaculatus* Pang & Mao, 1979: 55; Cao and Xiao 1984: 98; Cao et al. 1992: 133; Pang 1998: 186; Kovář 2007: 73, 575; Ren et al. 2009: 110. Synonymized by Kovář 2007: 73.

**Distribution.** China (Yunnan); Laos (Mouhot).

***Aspidimerus nigritus*** (Pang & Mao, 1979)

*Cryptogonus nigritus* Pang & Mao, 1979: 61; Cao et al. 1992: 138.

*Aspidimerus dongpaoensis* Hoàng, 1982: 165. Synonymized by Kovář 2007: 73.

*Aspidimerus nigritus* (Pang & Mao): Kovář 2007: 71, 73, 575; Ren et al. 2009: 108. Combined by Kovář 2007: 71.

**Distribution.** China (Yunnan); Vietnam.

***Aspidimerus nigrovittatus*** Motschulsky, 1866

*Aspidimerus nigrovittatus* Motschulsky, 1866: 424; Crotch 1874: 202; Weise 1900: 428; Korschefsky 1931: 172; Poorani 2002: 343.

**Distribution.** Sri Lanka.

***Aspidimerus ruficrus*** Gorham, 1895

*Aspidimerus ruficrus* Gorham, 1895: 690; Korschefsky 1931: 173; Kapur 1948: 83; Pang and Mao 1979: 55; Cao and Xiao 1984: 98; Cao et al. 1992: 134; Pang 1998: 186; Kovář 2007: 575; Ren et al. 2009: 108; Poorani 2002: 343.

*Cryptogonus blandus* Mader, 1954: 130; Liu 1963: 84.

*Aspidimerus blandus* (Mader, 1954): Kovář 2007: 73. Combined by Kovář 2007: 73. syn. n.

**Distribution.** China (Sichuan, Yunnan); Vietnam; Burma.

***Aspidimerus spencii*** Mulsant, 1850

*Aspidimerus spencii* Mulsant, 1850: 944; Crotch 1874: 202; Weise 1885: 232; Korschefsky 1931: 173; Kapur 1948: 83; Poorani 2002: 343.

**Distribution.** India; Burma.

***Aspidimerus zhenkangicus*** Huo & Ren, sp. n.

*Aspidimerus zhenkangicus* Huo & Ren, sp. n.

**Distribution.** China (Yunnan).

## Supplementary Material

XML Treatment for Aspidimerus

XML Treatment for Aspidimerus nigritus

XML Treatment for Aspidimerus esakii

XML Treatment for Aspidimerus menglensis

XML Treatment for Aspidimerus guangxiensis

XML Treatment for Aspidimerus matsumurai

XML Treatment for Aspidimerus kabakovi

XML Treatment for Aspidimerus decemmaculatus

XML Treatment for Aspidimerus mouhoti

XML Treatment for Aspidimerus zhenkangicus

XML Treatment for Aspidimerus ruficrus
